# An immunocompromised BALB/c mouse model for respiratory syncytial virus infection

**DOI:** 10.1186/1743-422X-2-3

**Published:** 2005-02-08

**Authors:** Xiaoyuan Kong, Gary R Hellermann, Geoff Patton, Mukesh Kumar, Aruna Behera, Timothy S Randall, Jian Zhang, Richard F Lockey, Shyam S Mohapatra

**Affiliations:** 1 Department of Internal Medicine, Division of Allergy and Immunology, Joy McCann Culverhouse Airway Disease Research Center, University of South Florida College of Medicine USA; 2 James A. Haley VA Hospital, Tampa, FL, USA

## Abstract

**Background:**

Respiratory syncytial virus (RSV) infection causes bronchiolitis in infants and children, which can be fatal, especially in immunocompromised patients. The BALB/c mouse, currently used as a model for studying RSV immunopathology, is semi-permissive to the virus. A mouse model that more closely mimics human RSV infection is needed. Since immunocompromised conditions increase risk of RSV infection, the possibility of enhancing RSV infection in the BALB/c mouse by pretreatment with cyclophosphamide was examined in this study. BALB/c mice were treated with cyclophosphamide (CYP) and five days later, they were infected with RSV intranasally. Pulmonary RSV titers, inflammation and airway hyperresponsiveness were measured five days after infection.

**Results:**

CYP-treated mice show higher RSV titers in their lungs of than the untreated mice. Also, a decreased percentage of macrophages and an increased number of lymphocytes and neutrophils were present in the BAL of CYP-treated mice compared to controls. The CYP-treated group also exhibited augmented bronchoalveolar and interstitial pulmonary inflammation. The increased RSV infection in CYP-treated mice was accompanied by elevated expression of IL-10, IL-12 and IFN-γ mRNAs and proteins compared to controls. Examination of CYP-treated mice before RSV infection showed that CYP treatment significantly decreased both IFN-γ and IL-12 expression.

**Conclusions:**

These results demonstrate that CYP-treated BALB/c mice provide a better model for studying RSV immunopathology and that decreased production of IL-12 and IFN-γ are important determinants of susceptibility to RSV infection.

## Introduction

Respiratory syncytial virus (RSV) is an important respiratory pathogen that produces an annual worldwide epidemic of respiratory illness primarily in children, but also in the elderly [1,2]. In the USA alone, RSV infection of children causes about 100,000 hospitalizations and 4,500 deaths annually (MMWR, 1996). RSV commonly precipitates bronchiolitis and exacerbates asthma but is also associated with severe life threatening respiratory infections in individuals with coronary artery disease or who are immunocompromised [3-6]. At the molecular level, RSV infection up-regulates the expression of several cytokines and chemokines, such as IL-1β, IL-6, IL-8, TNF-α, MIP1α, RANTES, and the adhesion molecule ICAM-1, ET-1, LTB4 and LTC4/D4/E4 [7-13]. Furthermore, elevated levels of cytokines and chemokines have been found in the nasal secretions of naturally RSV-infected children and of artificially-infected adults [14-17]. Defects in IL-12 and IFN-α production have been associated with severe RSV disease [18].

Despite progress in our understanding of immunopathology, the lack of a suitable animal model, with pathophysiology similar to humans, allowing appropriate virology, immunology, pathology and toxicology testing, has hindered the development of prophylactic and therapeutic interventions against RSV infection [19]. The pathology of RSV infection has been examined in a number of animals including primates, cotton rats, mice, calves, guinea pigs, ferrets and hamsters [19]. The choice of an experimental model is governed by the specific manifestation of the disease. The development of multiple animal models reflects the multifaceted nature of human RSV disease, in which clinical manifestations and sequelae depend upon age, genetic makeup, immunologic status and concurrent disease status of the individual [19]. Currently there is no single animal model that duplicates all forms of RSV disease. While cotton rats provide a good model for toxicologic evaluations, mice are considered advantageous for immunology and vaccine development. Furthermore, in mice the importance of IFN-γ, IL-6, IL-10 and IL-13 has been described [20-24]. The mouse provides an excellent model for human RSV infection because of the following: (a) the mouse is the best-characterized animal model, and experiments can be performed in this model in a cost- and time-effective manner, (b) a wide array of immunological reagents is available for studies in this model, and (c) the A2 strain of human RSV administered intranasally readily infects lungs of mice, and exhibits a time course of infection, pathology and resolution similar to that seen in humans [25]. Treatment of mice with the anti-RSV compound ribavirin decreases RSV titers in the lungs [26]. Depending upon the amount of RSV administered, the illness in micemay range from mild pneumonitis of the lung to weight loss [27].

Healthy BALB/c mice are semi-permissive to RSV and develop only limited inflammation and airway reactivity. Based on the reports that deficiency in IL-12 and IFN production increases severity of RSV disease, we hypothesized that rendering mice immunocompromised, will improve permissiveness to RSV and provide a better model for RSV infection. To test this hypothesis, BALB/c were treated with cyclophosphamide, infected with RSV, and characterized in terms of viral infectivity and pathology, immunology, and immunohistology. The results show that cyclophosphamide temporarily decreases IL-12 production and thus augments viral replication and the immunopathology of RSV disease.

## Materials and Methods

### Animals

Female six-week old BALB/c mice were purchased from Jackson Laboratory (Bar Harbor, ME) and maintained in a pathogen-free environment. All procedures were reviewed and approved by the University of South Florida Committee on Animal Research.

### Cyclophosphamide treatment

Cyclophosphamide (CYP; Sigma, St. Louis, MO) was administered to mice intraperitoneally (i.p.) at a single dose of 100 mg per kg five days prior to RSV infection.

### RSV infection, weight determination and tissue collection

The A2 strain of human RSV (American Type Culture Collection, Manassas, VA) was propagated in Hep-2 cells (ATCC) in a monolayer culture as previously described (Behera et al., 1998). Mice were infected intranasally with 5 × 10^5 ^PFU of RSV in a volume of 50 μl five days after treatment with CYP. One set of animals was monitored for weight loss at days 5, 10, 15 and 22 following CYP treatment (0, 5, 10 and 17 days after RSV infection). A second set of animals was sacrificed five days after infection and their lungs were removed for determination of RSV titers, cytokine levels and histopathology.

#### RSV plaque assay

HEp-2 cells (5 × 10^5^/well) in 6-well plates were infected with 5 × 10^5 ^pfu RSV per well for 2 hours at 37°C. The RSV was removed and the wells were overlaid with 1.5 ml of growth medium containing 0.8% methylcellulose. The cells were then incubated at 37°C for 72 hours, after which the overlay was removed. Following incubation, the cells were fixed in cold 80% methanol for 3 hours, blocked with 1 % horse serum in PBS at 37°C for 30 min, then incubated with anti-RSV monoclonal antibody (NCL-RSV 3, Vector Laboratories, Burlingame, CA) diluted 1:400 for 1 hour at 37°C. Secondary antibody staining and substrate reactions were performed using the Vectastain ABC Kit (Vector Laboratories) and diaminobenzidine in H_2_O_2 _(Pierce, Rockford, IL) was used as a chromagen. The plaques were enumerated by microscopy and the results were expressed as mean ± standard error of the mean.

### Determination of airway hyperresponsiveness (AHR)

AHR was measured in unrestrained mice using a whole body plethysmograph (Buxco, Troy, NY), as previously described (Schwarze et al., 1997) and expressed as enhanced pause (Penh). Groups of mice (n = 4) were exposed for 5 min to nebulized PBS and subsequently to increasing concentrations (6, 12, 25 and 50 mg/ml) of nebulized methacholine (MCh; Sigma, St, Louis, MO) in PBS using an ultrasonic nebulizer. After nebulization, recordings were taken for 5 minutes. Penh values were averaged and expressed as a percentage of baseline Penh values obtained following PBS exposure.

### Immunohistochemical analysis

Mouse lungs were rinsed with intratracheal injections of PBS then perfused with 10 % neutral buffered formalin. Lungs were removed, paraffin-embedded, sectioned at 20 μm and stained with hematoxylin and eosin (H & E). A semi-quantitative evaluation of inflammatory cells in the lung sections was performed as previously described (Kumar et al., 1999). Whole lung homogenates were prepared using a TissueMizer and assayed for cytokines IL-10, IL-12 and IFN-γ by ELISA (R & D Systems, Minneapolis MN), following the manufacturer's directions. The results are expressed as cytokine amount in picograms per gram of lung (pg/g).

### Detection of RSV and cytokines in the lungs by RT-PCR

Total cellular RNA was isolated from lung tissue using TRIZOL reagent (Life Technologies, Gaithersburg, MD). Forward and reverse primers used were as follows: RSV-N forward: 5'-GCG ATG TCT AGG TTA GGA AGA A-3'; reverse: 5'-GCT ATG TCC TTG GGT AGT AAG CCT-3'; mouse IFN-γ Forward: 5'-GCT CTG AGA CAA TGA ACG CT-3'; reverse: 5'-AAA GAG ATA ATC TGG CTG TGC-3'; mouse IL-10 forward: 5'-GGA CTT TAA GGG TTA CTT GGG TTG CC-3'; reverse: 5'-CAT TTT GAT CAT CAT GTA TGC TTC T-3'; mouse IL-12 forward: 5'-CAG TAC ACC TGC CAC AAA GGA -3'; reverse: 5'-GTG TGA CCT TCT CTG CAG ACA -3' and β-actin forward: 5'-GAC ATG GAG AAG ATC TGG CAC-3'; reverse: 5'-TCC AGA CGC AGG ATG GCG TGA -3'. All PCRs were denatured at 95°C for 1 min, annealed at 56°C for 30 sec, and extended at 72°C for 1 min for 25–35 cycles. All amplifications were done in triplicate and repeated three times. The PCR products were separated by agarose gel electrophoresis and quantified using Advanced Quantifier Software (BioImage, Ann Arbor, MI).

### Cell enumeration of bronchoalveolar lavage fluid

Bronchoalveolar lavage (BAL) fluid was collected and differential cell counts were performed as previously described (Kumar et al., 1999). Briefly, BAL was centrifuged and the cell pellet was suspended in 200 μl of PBS and counted using a hemocytometer. The cell suspensions were then centrifuged onto glass slides using a cytospin centrifuge at 1000 rpm for 5 min at room temperature. Cytocentrifuged cells were air dried and stained with a modified Wright's stain (Leukostat, Fisher Scientific, Atlanta, GA) which allows differential counting of monocytes and lymphocytes. At least 300 cells per sample were counted by direct microscopic observation.

### Statistical analysis

Values for all measurements were expressed as mean ± SD or SEM. The data were analyzed by ANOVA. Paired and unpaired results were compared by a Wilcoxon rank sum test or Mann-Whitney test respectively. Differences between groups were considered significant at *p *< 0.05.

## Results

### Cyclophosphamide treatment augments RSV infection in mice

**Figure 1:**
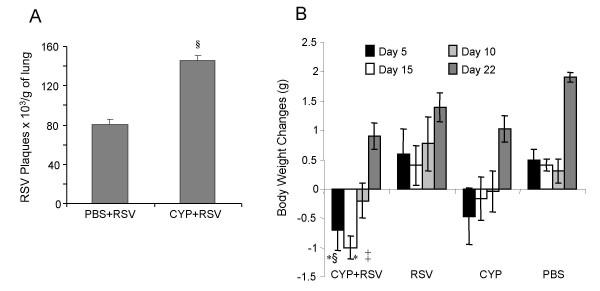
**(A) CYP increases RSV titer in the lungs of BALB/c mice. **Mice were treated with CYP (100 mg/kg, i.p.) or PBS and 5 days later infected with RSV (50 μl i.n. twice, 10^6 ^PFU/ mouse). Animals were sacrificed on day 4 and RSV titers were measured in whole lung homogenates by RSV plaque assay. (n = 4 for each group; § P < 0.01 vs PBS group). **(B) Cyclophosphamide affects body weight. **Mice (n = 4) were infected with RSV alone or were treated with CYP (100 mg/kg i.p.) prior to infection. Body weights were measured on day 1, 5, 10, 15, and 22 after treatment. Bars represent means ± SEM. (* P < 0.05; †. P < 0.01 vs RSV); ‡ P < 0.05; § P < 0.01 vs control).

Groups of mice were injected i.p. with a single dose of CYP or PBS and five days later infected with RSV. Five days after infection, RSV titers in one group of mice were measured by plaque assay of lung homogenates (Fig. 1A). The mice pretreated with CYP produced significantly more (*p *< 0.01) RSV plaques compared to the PBS control group. Weight loss is a clinical correlate of RSV infection, therefore weights were measured in a parallel group of mice on day 5, 10, 15, and 22 after CYP treatment (day 0, 5, 10 and 17 after RSV infection) (Fig. 1B). CYP treatment alone resulted in a weight loss or reduced weight gain compared to PBS, but the RSV-infected, CYP-treated mice lost significantly more weight than those exposed to RSV alone p < 0.05; †.p < 0.01(vs. RSV) or PBS (p < 0.01 vs. Control). Pretreatment with CYP resulted in increased weight loss in the RSV-infected mice through day 15 and reduced weight gain at day 22 indicating that cyclophosphamide treatment exacerbated the pathology of RSV infection.

### Cyclophosphamide pretreatment increases RSV-inducible lung inflammation

**Figure 2:**
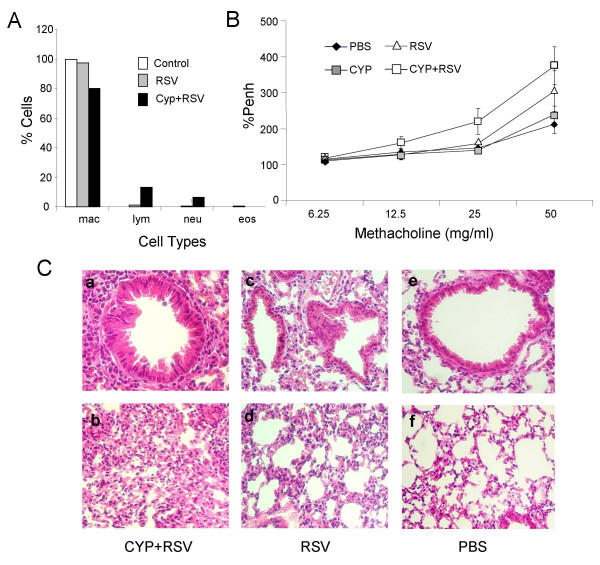
**(A) BAL cell differential of RSV-infected mice**. Mice were treated with cyclophosphamide (CYP) or vehicle 5 days before infection with RSV. Animals were sacrificed on day 4 postinfection and BAL was performed. Following cytocentrifugation, BAL cells were stained with Leukostat and counted from 4 different slides from each group in a blinded fashion. Cell counts as percentage of total were plotted. **(B) Measurement of airway hyperrresponsiveness (AHR)**. Mice treated as above were tested for AHR by methacholine challenge in a plethysmograph. AHR is expressed as PENH, percent of control. **(C) Lung histopathology. **Mice were infected with RSV alone **(C and D) **or treated with cyclophosphamide **(A and B) **prior to RSV infection. The third group of mice was not exposed to RSV **(E and F). **Animals were sacrificed on day 5 and their lungs removed and sectioned. Paraffin-embedded lung sections were stained with hematoxylin-eosin.

To examine whether CYP treatment increases inflammatory effects in the lungs of RSV-infected mice, we determined airway hyperresponsiveness (AHR), cellular infiltration into the lung and lung histopathology. Groups of BALB/c mice either infected with RSV alone or treated with cyclophosphamide (CYP) prior to infection were lavaged and cells in the fluid were centrifuged onto slides. BAL cells were stained with Leukostat. Cells were counted from 4 different slides from each group in a blinded fashion. Cell counts were plotted as percentage of total cells (Fig. 2A). There was a decrease in the number of macrophages and increases in lymphocyte and neutrophil numbers following RSV infection that was enhanced by prior treatment with CYP. To analyze the extent of lung pathology, lungs were paraffin embedded, sectioned and stained with hemotoxylin-eosin (HE). The lung sections from RSV-infected, CYP-treated mice (Fig. 2C, a & b) showed significantly greater inflammation than lungs from mice given RSV alone (Fig. 2C, c & d). The RSV-infected groups showed greater inflammation than uninfected control mice (Fig. 2C e & f).

### Increased RSV infection in CYP-treated BALB/c mice is associated with increased production of immunoregulatory cytokines

**Figure 3:**
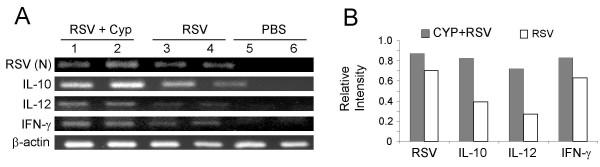
**Detection of RSV and cytokines in the lungs of BALB/c mice. (A) **RSV-N and IL-10, IL-12, IFN-γ and β-actin were checked by RT-PCR. Mice were infected with RSV alone or treated with CYP prior to infection. The third group was uninfected (PBS) as control. Animals were sacrificed on day 5, their lungs removed and RNA was isolated and used in RT-PCR assay. **(B) Densitometric analysis of the band densities from part A. **Relative intensity refers to the ratio of the intensity of each cDNA product to that of β-actin.

**Figure 4:**
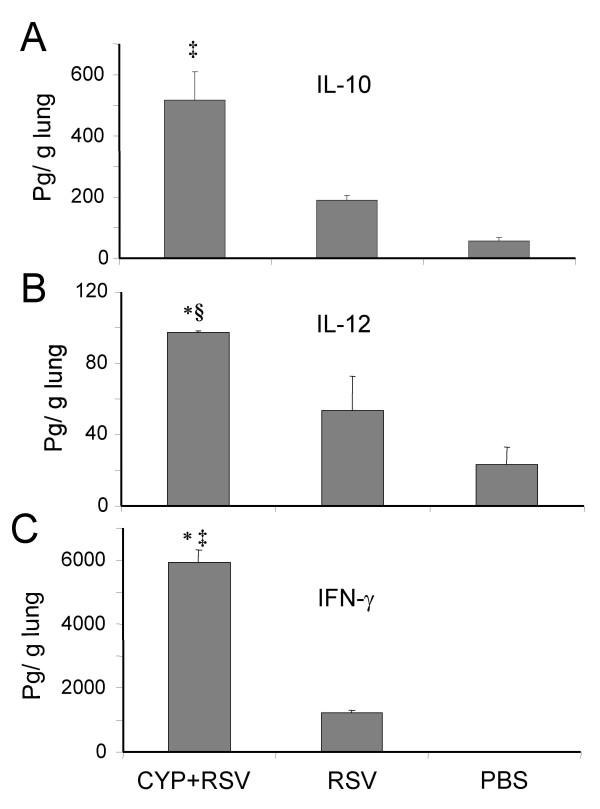
**CYP-treated BALB/c mice produce higher levels of Th1 and Th2 cytokines**. Mice were infected with RSV alone or were treated with cyclophosphamide prior to infection. The third group of mice received no virus (PBS only) as control. Animals were sacrificed on day 5 and their lungs removed. Whole lung homogenates were prepared and cyrtokines were measured by ELISA. Results are given as mean ± SEM (n = 4 for each group). **(A) **CYP-pretreated mice produce higher IL-10 in the lungs. (‡ P < 0.05 vs. PBS). **(B) **IL-12 was higher in CYP-treated mice. (§ P < 0.01 vs. PBS. * P < 0.05 vs. RSV). **(C) **IFN-γ was higher in CYP-treated mice. (* P < 0.05 vs. RSV; ‡ P < 0.05 vs. PBS).

To examine the cytokine profile in lungs from RSV-infected mice with or without CYP treatment, the gene expression of IL-10, IFN-γ and IL-12 was measured by RT-PCR. Gel profiles and densitometric analyses are shown in Fig. 3A and 3B. The results show that mice infected with RSV after CYP treatment have increased mRNA expression for all of three cytokines compared to control mice or mice infected with RSV alone. To determine whether mice treated with CYP and infected with RSV do produce more of these proteins, cytokines were measured by ELISA on homogenates prepared from whole lungs. IL-10 (p < 0.05 vs. PBS), IL-12 (p < 0.05 vs. RSV) and IFN-γ (p < 0.05 vs. PBS) levels were significantly higher in the CYP-treated group than the control group (Fig. 4A–C).

### Cyclophosphamide treatment causes a transient reduction in IL-12 and IFN-γ expression

**Figure 5:**
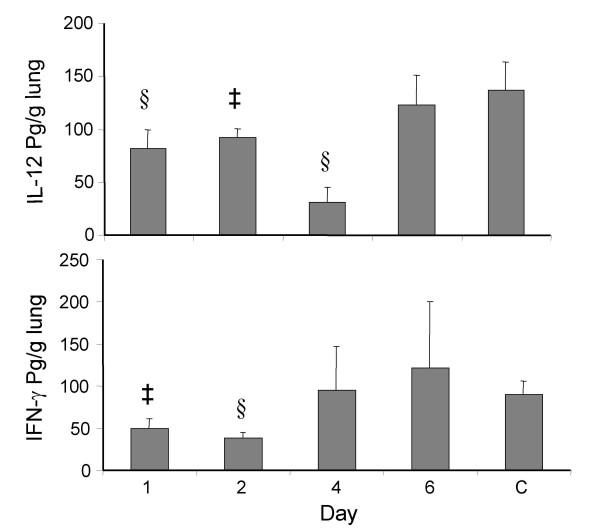
**Changes in IL-12 and IFN-γ levels over time in CYP-treated mice. **Mice were treated with cyclophosphamide at 100 mg/kg i.p. Animals were sacrificed on day 1, 2, 4, 6 after treatment and their lungs removed. Whole lung homogenates were prepared and IL-12 **(A) **and IFN-γ **(B) **were measured by ELISA. Untreated mice were used as control. Results are shown as mean ± SEM (n = 2; ‡ P < 0.05; § P < 0.01 vs. control).

To examine the mechanism underlying the increased RSV infection in CYP-treated animals, the levels of two cytokines that exert antiviral activity, IL-12 and IFN-γ was measured after treatment with CYP (Fig. 5). Mice were sacrificed on days 1, 2, 4, 6 after treatment and IL-12 and IFN-γ protein levels were measured in whole lung homogenates by ELISA. Untreated BALB/c mice were used as controls. Treatment with CYP gradually decreased both IL-12 (p < 0.05) and IFN-γ (p < 0.01 vs. Control) until day 4. These results show that decreased production of IL-12 and IFN-γ may play a role in the observed increase in RSV infection in CYP-treated mice.

## Discussion

The main focus of this study has been to establish and characterize an immunocompromised mouse model for studying RSV infection. Mice are only semi-permissive to RSV infection, yet can serve as a useful model for immunological studies. Compared to the traditional BALB/c mouse model, the use of cyclophosphamide to create an immunocompromised condition provides an effective means of augmenting RSV replication and disease. Pretreatment of BALB/c mice with CYP results in RSV titers in the lungs of these immunocompromised mice that are increased significantly compared to the group infected with RSV without cyclophosphamide treatment. These results are consistent with an earlier study in the cotton rat model [28]. In another study, a high titer RSV inoculum (10^7 ^PFU/ml) was administered intranasally to old mice and resulted in clinical illness and appreciable pathology in the lung [25], while mice inoculated with 10^6 ^PFU/ml, or less, did not exhibit symptoms of illness. Most studies using mice as models employ lower doses of RSV because RSV infection in humans, which induces a pneumonia-like pulmonary inflammation, occurs typically at sub-clinical RSV doses. The loss of body weight in CYP-treated mice following RSV infection compared to untreated control mice confirmed that CYP-treatment increased the susceptibility to RSV infection at lower inocula. This finding that permissiveness to RSV can be augmented by rendering mice immunocompromised is significant, as it increases the utility of the mouse model for RSV infection.

Consistent with increased RSV infection, the cellular population in BAL fluid was altered. Especially significant are the increases in lymphocytes and neutrophils in CYP-treated mice compared to controls. This data is in agreement with previous reports showing that RSV infection increased lymphocyte infiltration in the lung [2]. Along with increased cellular infiltration, lung pathology, particularly epithelial denudation and goblet cell hyperplasia, is also markedly increased.

Expression of IL-10, IL-12 and IFN-γ was examined to determine if RSV-induced changes in the levels of these cytokines in the lungs of CYP-treated mice played a role in the increased lung immunopathology. RSV-infected CYP-treated mice exhibited significantly increased expression of these cytokines at the protein and mRNA level in agreement with previous observations that RSV infection induced enhanced expression of Th1 and Th2 cytokines [22,29]. Other studies have shown that IFN-γ can induce production of IL-12 in a self-activating loop, by activating macrophages which produce IL-12 [30].

Although CYP treatment is known to induce an immunosuppressed condition, the mechanism is unclear. The results of cytokine analysis on days 1 to 5 after cyclophosphamide treatment indicated that IL-12 and IFN-γ were reduced and the reduction was highest on days four and two, respectively. These results suggest that the ability of cells to produce these cytokines at the time of RSV infection is an important determinant of the magnitude of infection in terms of increased RSV replication and titer. Impairment in IL-12 and IFN-γ production at the key moment of acute infection leads to rapid viral replication and subsequent pathology. In a previous report we demonstrated the importance of IFN-γ by artificially increasing IFN-γ levels and showing that viral titers were decreased because of the induction of 2'-5'oligoadenylate synthetase which activates RNase L to degrade viral RNA [31]. Also, studies in humans have suggested that individuals lacking IFN-α or IL-12 are at a higher risk of severe RSV disease. Thus, down-regulation of the production of these cytokines is a likely factor underlying the observed enhancement of RSV infection by cyclophosphamide treatment.

In conclusion, the results of this study demonstrate that cyclophosphamide treatment of BALB/c mice renders them more susceptible to RSV infection as revealed by increased RSV titers in the lung and decreased body weight. The mechanism of this increase in infection involves transient down regulation of IFN-γ and IL-12 induced by cyclophosphamide treatment.

## Competing Interests

The author(s) declare that they have no competing interests.

## Authors' Contributions

XK, BAL and cell enumeration; GH, data analysis; GP, AHR, RT-PCR; MK, RSV infection and assay, tissue collection, RT-PCR; AB, cyclophosphamide treatment, tissue collection, ELISAs; TSR, immunohistochemistry; JZ, cell culture and virus preparation; RFL, experimental design and analysis; SSM, project design, experimental analysis and data interpretation.
